# Prospective Comparative Evaluation of Stone Clearance and Complications in Renal Stones Measuring 2 to 3.5 Centimeters: Percutaneous Nephrolithotomy (PCNL) versus Retrograde Intrarenal Surgery (RIRS)

**DOI:** 10.7759/cureus.93302

**Published:** 2025-09-26

**Authors:** Javeria Hayat Khan, Sajid Malik, Yassar Hussain Patujo, Shakeel Haseeb Uddin Siddique, Muhammad Moosa, Muhammad Tayyab Khan, Ayesha Masood

**Affiliations:** 1 Urology, Pakistan Atomic Energy Commission General Hospital, Islamabad, PAK; 2 Urology, Jinnah Teaching Hospital Peshawar, Peshawar, PAK; 3 Urology, Chandka Medical College, Larkana, PAK; 4 Urology, The Kidney Center, Karachi, PAK; 5 Urology, Hayatabad Medical Complex Peshawar, Peshawar, PAK; 6 General Surgery, Hayatabad Medical Complex Peshawar, Peshawar, PAK; 7 Perfusionist, Dow University of Health Sciences, Karachi, PAK

**Keywords:** endourology, nephrolithiasis, percutaneous nephrolithotomy (pcnl), renal stones, retrograde intrarenal surgery (rirs), stone clearance, urolithiasis

## Abstract

Background

Percutaneous nephrolithotomy (PCNL) and retrograde intrarenal surgery (RIRS) are the two most commonly used minimally invasive surgical techniques for renal stone disease, which is a major worldwide health burden. Because it is less intrusive, RIRS has become more common even though PCNL is still the gold standard for big renal stones. Clinical decision-making regarding the best intervention requires the use of comparative evidence.

Objective

To evaluate the perioperative results and stone-free rates of PCNL and RIRS in patients with kidney stones.

Methodology

In this prospective randomized controlled trial, patients with kidney stones (2-3.5 cm; n=440) were split evenly between two groups: PCNL (n=220) and RIRS (n=220). The following outcomes were evaluated: perioperative problems, hospital stay, operation time, stone-free rate, and auxiliary procedures. Independent t-tests for continuous variables and chi-square tests for categorical variables were used in the statistical analysis; p<0.05 was deemed significant.

Results

The PCNL group had a significantly higher stone-free rate than the RIRS group (90.0% vs. 80.0%, χ²=8.57, p=0.003). The mean operative time was longer in the PCNL group compared to RIRS (92.4 ± 18.5 vs. 78.6 ± 15.3 min, t=8.42, p<0.001). The mean hospital stay was also longer for PCNL (3.6 ± 1.2 vs. 1.8 ± 0.7 days, t=18.7, p<0.001). Patients undergoing PCNL experienced greater intraoperative bleeding (17.3% vs. 2.7%, χ²=26.9, p<0.001) and a higher mean hemoglobin drop (1.6 ± 0.8 vs. 0.7 ± 0.5 g/dl, t=13.1, p<0.001). Auxiliary procedure rates were higher in the RIRS group (20.0% vs. 10.0%, χ²=8.57, p=0.003).

Conclusion

Although PCNL provides better stone removal than RIRS, it is linked to longer operating times, longer hospital stays, and higher incidence of complications. Individualized treatment should be chosen according to the patient's and the stone's characteristics.

## Introduction

Kidney stone disease (nephrolithiasis) is a widespread urological condition, affecting between 1% and 15% of the global population during their lifetime [1]. In 2015 alone, there were approximately 22.1 million cases worldwide, with an estimated 16,100 related deaths [2]. The incidence of nephrolithiasis continues to rise, driven by factors such as dehydration, sedentary lifestyles, high sodium intake, and dietary changes [3].

In Pakistan, in the so-called "stone belt" region, the burden of kidney stones is notably high [4]. A study from Khyber Pakhtunkhwa revealed a male predominance (53.3%) and a peak incidence among individuals aged 31 to 45 years, with a significant link to low fluid intake (mean daily intake <1.5 L in 68% of patients), frequent tea consumption (reported in 72%), and high dietary sodium [5]. Other regions, like Rawalpindi, reflected similar trends, with younger adults, lower socioeconomic status, and inadequate hydration emerging as major risk factors.

The size, location, and patient-specific characteristics of renal stones play a major role in therapy decisions. Because of its high stone-free rates, percutaneous nephrolithotomy (PCNL) has long been regarded as the gold standard for stones larger than 2 cm [6]. This >2 cm threshold is widely accepted because stone clearance rates with less invasive methods such as shockwave lithotripsy or single-session ureteroscopy decrease significantly beyond this size, whereas PCNL maintains consistently high success [6]. Retrograde intrarenal surgery (RIRS), on the other hand, has become a very attractive minimally invasive substitute [7]. Due to technological advancements, RIRS can now attain cumulative stone-free rates of 77% to 93% for stones larger than 2 cm, especially when multiple sessions are used [8].

Meta-analyses offered mixed results; one study found no significant difference in stone-free rates between RIRS and PCNL for stones >2 cm, though RIRS resulted in shorter hospital stays and less bleeding, despite longer operative times [8]. Conversely, other data suggest superior stone-free outcomes with PCNL; one meta-analysis reported a relative risk (RR) favoring PCNL (RR ~1.11), though with heterogeneity across studies [9].

However, there is a lack of large, prospective, comparative studies from the South Asian "stone belt" region that directly compare PCNL and RIRS for stones specifically in the 2-3.5 cm range. The high metabolic burden of kidney stones in Pakistan, coupled with limitations in healthcare access and resources, underscores the importance of identifying the most effective and safest treatment modality for this patient population.

Therefore, this study was designed with the hypothesis that PCNL would achieve higher stone-free rates but at the cost of longer operative time and higher complication rates compared to RIRS. By directly comparing outcomes under standardized operating conditions, this research seeks to provide evidence-based guidance for clinicians managing large renal stones, ultimately helping optimize patient outcomes and resource allocation.

## Materials and methods

This prospective comparative study was conducted at the Department of Urology and Renal Transplant Surgery at the Pakistan Atomic Energy Commission General Hospital (PAECGH), Islamabad. The study spanned one year, from 1st December 2024 to 30th November 2025. Ethical clearance was obtained from the Institutional Review Board (IRB) of PAECGH before commencement (approval no. PGHI-IRB-(DME)-RCD_06_059). Written informed consent was obtained from all patients before enrollment in the study.

The study population comprised patients presenting with renal stones measuring 2 to 3.5 cm, diagnosed by non-contrast CT Kidneys, Ureters, and Bladder (KUB) or intravenous urography (IVU). While CT was considered the reference standard for stone burden assessment, IVU findings were permitted if consistent with clinical and ultrasound findings; any discrepancies between modalities were resolved in favor of CT. All eligible patients who met the inclusion criteria during the study period were invited to participate in the sampling process, which was carried out using a non-probability sequential technique. Patients with renal stones within the designated size range and who were between the ages of 18 and 65, regardless of gender, were included.

Exclusion criteria consisted of patients with solitary kidneys, congenital or acquired renal anomalies, uncorrected bleeding disorders, active urinary tract infections, pregnancy, or severe cardiopulmonary comorbidities precluding anesthesia. For the latter, “severe” was operationalized as New York Heart Association (NYHA) class III-IV heart failure, uncontrolled arrhythmias, severe chronic obstructive pulmonary disease (Forced Expiratory Volume in 1 Second or FEV1 <50% predicted), or any condition judged by the anesthesiologist to significantly increase perioperative risk.

The sample size was calculated using OpenEpi software (Dean AG, Sullivan KM, Soe MM. OpenEpi: Open Source Epidemiologic Statistics for Public Health, Version. www.OpenEpi.com, updated 2013/04/06) based on a two-proportion comparison (Fleiss with continuity correction). For a 95% confidence level, 80% power, and equal allocation, with expected stone-free rates of 90% for PCNL and 80% for RIRS from previous literature, a minimum of 199 patients per group was required [10]. To compensate for potential attrition and incomplete follow-up, the final sample size was set at 220 patients per group, making a total of 440 participants.

Demographic details, including age, gender, BMI, socioeconomic status, and clinical information such as stone size, laterality, and comorbidities, were documented on a structured proforma. All participants underwent baseline investigations, including complete blood counts, renal function tests, coagulation profile, urinalysis, urine culture, ECG, chest X-ray, and ultrasonography. Stone size and burden were primarily assessed with non-contrast CT KUB before intervention.

Depending on the surgical technique used, patients were divided into two groups. Using fluoroscopic guidance to gain entry, Group A received PCNL while in the prone position and under general anesthesia. Nephroscope (Karl Storz GmbH & Co. KG, Tuttlingen, Germany) insertion came after tract dilatation, and either pneumatic or ultrasonic lithotripsy was used to fracture the stone. Under general anesthesia, Group B received RIRS using a flexible ureterorenoscope that was inserted through an access sheath. Holmium: yttrium-aluminum-garnet (YAG) laser (MIKAS Corporation, Rawalpindi, Pakistan) lithotripsy was used for fragmentation, and particles less than 2 mm were regarded as clinically unimportant remaining fragments.

The main end measure was stone clearance, which was determined at four weeks by non-contrast CT KUB (preferred for accuracy) or ultrasonography when CT was contraindicated (e.g., renal impairment, pregnancy concerns, or patient refusal due to radiation exposure). We acknowledge that ultrasonography is less sensitive for small residual fragments; however, its use in selected cases was considered acceptable for ethical and safety reasons. Stone clearance was defined as the absence of detectable stones or clinically insignificant fragments <2 mm.

The Clavien-Dindo system [11] was used to classify postoperative problems, such as bleeding, fever, urinary tract infection, or ureteric injury, as well as secondary outcomes including operating time, duration of hospital stay, and need for auxiliary operations. Patients were monitored for early problems 48 hours after surgery and for a final evaluation of stone clearance at four weeks.

IBM SPSS Statistics for Windows, Version 25 (Released 2017; IBM Corp., Armonk, New York, United States) was used to evaluate all of the data. While qualitative factors like gender, stone-free status, and complications were given as frequencies and percentages, quantitative variables like age, BMI, stone size, surgical time, and hospital stay were expressed as mean ± standard deviation. Continuous variables were compared using the independent t-test, while categorical variables were compared between the groups using the chi-square test. Statistical significance was defined as a p-value of less than 0.05.

## Results

The study comprised 440 patients in total, split evenly between the PCNL group (n=220) and the RIRS group (n=220). Table 1 displays the baseline demographic and clinical characteristics of the two groups.

**Table 1:** Baseline demographics of patients undergoing PCNL vs. RIRS PCNL: Percutaneous Nephrolithotomy; RIRS: Retrograde Intrarenal Surgery; Data are represented as Mean ± SD or n (%). Independent-samples t-test and Chi-square test applied. *p<0.05 is considered significant.

Variable	PCNL group (n=220)	RIRS group (n=220)	Test statistic	p-value
Age (years, Mean ± SD)	45.6 ± 11.2	44.8 ± 10.9	t = 0.74	0.459
Male participants	140 (63.6%)	134 (60.9%)	χ² = 0.33	0.564
Female participants	80 (36.4%)	86 (39.1%)		
BMI (kg/m², Mean ± SD)	27.1 ± 3.9	26.8 ± 4.1	t = 0.77	0.442
Laterality right	118 (53.6%)	110 (50.0%)	χ² = 0.61	0.436
Laterality Left	102 (46.4%)	110 (50.0%)		
Mean stone size (mm, Mean ± SD)	23.5 ± 5.8	18.9 ± 4.6	t = 9.48	<0.001*

The two cohorts were comparable at baseline, with no statistically significant difference between them in terms of age, gender distribution, BMI, or laterality of stone. However, the mean stone size was significantly larger in the PCNL group compared to the RIRS group (23.5 ± 5.8 mm vs. 18.9 ± 4.6 mm, p<0.001).

Table 2 summarizes the stone characteristics of the study participants.

**Table 2:** Characteristics of the kidney stones of the study participants (n=440) PCNL: Percutaneous Nephrolithotomy; RIRS: Retrograde Intrarenal Surgery (RIRS); Data are represented as n (%). Chi-square test applied. *p<0.05 is considered significant.

Variable	PCNL group (n=220)	RIRS group (n=220)	Test statistic	p-value
Staghorn calculi	42 (19.1%)	12 (5.5%)	χ² = 20.4	<0.001*
Multiple stones	80 (36.4%)	68 (30.9%)	χ² = 1.49	0.223
Lower pole stones	62 (28.2%)	58 (26.4%)	χ² = 0.15	0.696

Staghorn calculi were significantly more common in the PCNL group (19.1%) compared to the RIRS group (5.5%) (p<0.001). The prevalence of multiple stones and lower pole stones, however, was comparable between the two groups, with no statistically significant differences observed.

Table 3 highlights the operative and perioperative outcomes.

**Table 3:** Operative and perioperative outcomes of the study participants (n=440) PCNL: Percutaneous Nephrolithotomy; RIRS: Retrograde Intrarenal Surgery; Data are represented as Mean ± SD or n (%). Independent-samples t-test and Chi-square test applied. *p<0.05 is considered significant.

Variable	PCNL group (n=220)	RIRS group (n=220)	Test statistic	p-value
Mean operative time (min ± SD)	92.4 ± 18.5	78.6 ± 15.3	t = 8.42	<0.001*
Mean hospital stay (days ± SD)	3.6 ± 1.2	1.8 ± 0.7	t = 18.7	<0.001*
Stone-free rate (%)	198 (90.0%)	176 (80.0%)	χ² = 8.57	0.003*
Need for auxiliary procedure	22 (10.0%)	44 (20.0%)	χ² = 8.57	0.003*
Postoperative Fever	30 (13.6%)	18 (8.2%)	χ² = 3.07	0.080
Mean Hemoglobin Drop (g/dl ± SD)	1.6 ± 0.8	0.7 ± 0.5	t = 13.1	<0.001*

PCNL was associated with a significantly longer operative time (92.4 ± 18.5 min vs. 78.6 ± 15.3 min, p<0.001) and longer hospital stay (3.6 ± 1.2 days vs. 1.8 ± 0.7 days, p<0.001). However, the stone-free rate was significantly higher in the PCNL group (90.0% vs. 80.0%, p=0.003). The RIRS group demonstrated a higher need for auxiliary procedures (20.0% vs. 10.0%, p=0.003). Intraoperative hemoglobin drop was more pronounced in the PCNL group (1.6 ± 0.8 g/dl vs. 0.7 ± 0.5 g/dl, p<0.001).

Table 4 presents the postoperative complications using the Clavien-Dindo classification.

**Table 4:** Postoperative complications (Clavien-Dindo Classification) of the study participants (n=440) PCNL: Percutaneous Nephrolithotomy; RIRS: Retrograde Intrarenal Surgery; Data are represented as n (%). Chi-square test applied. p<0.05 is considered significant.

Complication Grade	PCNL group (n=220)	RIRS group (n=220)	Test Statistic	p-value
Grade I (minor)	26 (11.8%)	18 (8.2%)	χ² = 1.61	0.205
Grade II (infection)	22 (10.0%)	12 (5.5%)	χ² = 3.28	0.070
Grade III (re-intervention)	10 (4.5%)	8 (3.6%)	χ² = 0.21	0.644
Grade IV–V (severe/mortality)	2 (0.9%)	0 (0.0%)	χ² = 2.01	0.156

Overall complication rates were comparable between the groups, with no significant differences in minor complications (Grade I-II) or severe complications (Grade III-V). While severe complications were slightly more common in the PCNL group, this difference did not reach statistical significance (p=0.156). Importantly, no mortality was observed in either group.

Table 5 outlines the three-month follow-up outcomes.

**Table 5:** Three-month follow-up outcomes of the study participants (n=440) PCNL: Percutaneous Nephrolithotomy; RIRS: Retrograde Intrarenal Surgery; Data are represented as n (%). Chi-square test applied. *p<0.05 is considered significant.

Variable	PCNL group (n=220)	RIRS group (n=220)	Test statistic	p-value
Residual Fragment Recurrence	9 (4.1%)	21 (9.5%)	χ² = 4.35	0.037*
Re-intervention	8 (3.6%)	20 (9.1%)	χ² = 5.30	0.021*

Residual fragment recurrence was significantly higher in the RIRS group (9.5%) compared to the PCNL group (4.1%) (p=0.037). Similarly, re-intervention rates were significantly greater in the RIRS group (9.1%) compared to the PCNL group (3.6%) (p=0.021), indicating superior durability of outcomes with PCNL in the medium term.

## Discussion

In this prospective comparative study of 440 patients with renal stones measuring 2.0-3.5 cm, PCNL achieved a higher final stone-free rate than RIRS, while RIRS was associated with shorter hospital stay and lower intraoperative bleeding. These findings align closely with contemporary literature. PCNL generally offers superior single-session stone clearance for larger stones, while RIRS is less invasive with shorter recovery but may require more auxiliary procedures or staged sessions to reach comparable clearance [12,13,14]. In our center, conventional PCNL and standard flexible ureteroscopy with Holmium: YAG laser were utilized; mini-PCNL and high-power dusting lasers were not routinely used, which contextualizes the applicability of external evidence to our results.

Multiple meta-analyses and comparative studies support the higher efficacy (stone-free rate) of PCNL for larger renal stones. Kallidonis et al. (2020) summarized evidence showing that PCNL outperforms RIRS in stone-free rate for stones >2 cm, while RIRS is associated with reduced hospital stay and morbidity, mirroring our efficacy and morbidity trade-offs [15]. Similarly, Wang et al. (2021) reported significantly higher stone-free rates with PCNL, though with greater bleeding and longer hospitalization, consistent with the higher hemoglobin drop and inpatient stay we observed in the PCNL arm [16].

A 2022 meta-analysis including mini-PCNL series found superior stone-free rate for PCNL but higher complications and longer stay; RIRS offered improved safety and faster recovery but lower single-session clearance and higher auxiliary procedures [8,17]. Our data similarly demonstrate a higher auxiliary-procedure rate and residual fragment frequency in the RIRS cohort. Prospective cohort studies also support this duality: Ucer et al. (2021) observed higher transfusion and hospital stay with PCNL, and higher retreatment/auxiliary procedure rates with RIRS, paralleling our findings [18]. Liu et al. (2023) concluded that minimally invasive PCNL variants and conventional PCNL generally produce higher primary stone-free rates for larger stones, while RIRS provides lower major bleeding and shorter hospitalization, concordant with our outcomes [19].

Some studies indicate that modern RIRS technologies (high-power lasers, dusting techniques, improved access sheaths) or miniaturized PCNL approaches narrow the outcome differences. Lee et al. (2022) reported no significant difference in stone-free rates between tubeless mini-PCNL and RIRS in selected patients, underscoring how technique refinements can close the stone-free rate gap [20]. Qiu et al. (2024) found mini-PCNL provided higher stone-free rates in obese patients but with longer hospital stay and grade-I complications, highlighting the influence of patient selection and the PCNL variant [21].

Heterogeneity in stone burden, use of single versus staged procedures, and definitions of “stone-free” (CT vs ultrasound, clinically insignificant fragment thresholds) affect absolute stone-free rate estimates. Advances in endoscopic technology-suctioning access sheaths, high-power lasers, micro-PCNL devices-shift the comparative balance, sometimes making RIRS more competitive for larger stones. Our findings of higher auxiliary-procedure rates but acceptable cumulative stone-free rates after staged RIRS are consistent with these observations [22,23].

Differences between our results and particular studies may reflect stone complexity (higher staghorn/large-burden stones in our PCNL group), surgical expertise, and institutional protocols, as well as varying definitions of clinically insignificant residual fragments (<2 mm in our study vs <3-4 mm elsewhere) [17,19].

Clinically, for renal stones 2.0-3.5 cm, PCNL remains the preferred option when single-session clearance is prioritized, particularly for complex or staghorn stones. RIRS offers a valuable minimally invasive alternative for patients where bleeding risk, shorter hospitalization, or faster recovery is a priority, accepting potentially lower single-session SFR and the need for auxiliary procedures.

Strengths and Limitations

Strengths of this study include its prospective comparative design, large sample size, and standardized outcome measures. Limitations include its non-randomized design, introducing potential selection bias as the procedure choice depended on surgeon and patient preference despite baseline comparability; single-center setting limiting generalizability; short-term follow-up focusing on four-week stone-free rates rather than long-term recurrence or renal functional outcomes; and absence of cost-effectiveness and patient-reported outcome measures.

Future research should include randomized trials comparing modern RIRS (high-power laser dusting, suction ureteral access sheaths (UAS)) versus miniaturized PCNL for stones 2-3.5 cm, with long-term stone-free rates, recurrence, renal function, health economic analysis, and patient-centered outcomes. Stratification by stone complexity and integration of newer technologies (suctioning access sheaths, novel lasers, image-guided PCNL access) should be evaluated in pragmatic multicenter trials to determine if the historical stone-free rate gap can be further narrowed without increasing morbidity.

## Conclusions

This study's results provide valuable insights into the comparative benefits of PCNL and RIRS for renal stones. PCNL achieved higher stone clearance, particularly for larger or complex stones, albeit with longer hospital stays and greater perioperative morbidity. In contrast, RIRS was less invasive, associated with shorter hospitalization, and demonstrated a favorable safety profile, making it a suitable option for selected patient subgroups such as those with higher bleeding risk, comorbidities, or a preference for faster recovery. These findings underscore the importance of tailoring surgical decisions based on patient-specific factors, including stone size, location, comorbidities, and risk tolerance. While PCNL remains the standard for achieving maximal single-session stone clearance in larger or complex stones, ongoing technological advances in RIRS and miniaturized PCNL approaches may narrow this efficacy gap, offering increasingly viable alternatives in appropriately selected patients.
